# The road not taken: retreat and diverge in local search for simplified protein structure prediction

**DOI:** 10.1186/1471-2105-14-S2-S19

**Published:** 2013-01-21

**Authors:** Swakkhar Shatabda, MA Hakim Newton, Mahmood A Rashid, Duc Nghia Pham, Abdul Sattar

**Affiliations:** 1 Institute of Intelligent and Integrated Systems, Griffith University, Queensland, Australia; 2 Queensland Research Laboratory, National ICT of Australia

## Abstract

**Background:**

Given a protein's amino acid sequence, the protein structure prediction problem is to find a three dimensional structure that has the native energy level. For many decades, it has been one of the most challenging problems in computational biology. A simplified version of the problem is to find an on-lattice self-avoiding walk that minimizes the interaction energy among the amino acids. Local search methods have been preferably used in solving the protein structure prediction problem for their efficiency in finding very good solutions quickly. However, they suffer mainly from two problems: re-visitation and stagnancy.

**Results:**

In this paper, we present an efficient local search algorithm that deals with these two problems. During search, we select the best candidate at each iteration, but store the unexplored second best candidates in a set of elite conformations, and explore them whenever the search faces stagnation. Moreover, we propose a new non-isomorphic encoding for the protein conformations to store the conformations and to check similarity when applied with a memory based search. This new encoding helps eliminate conformations that are equivalent under rotation and translation, and thus results in better prevention of re-visitation.

**Conclusion:**

On standard benchmark proteins, our algorithm significantly outperforms the state-of-the art approaches for Hydrophobic-Polar energy models and Face Centered Cubic Lattice.

## Background

Proteins are the most important of all organ-isms present in the living cell. Given a protein's amino acid sequence, the protein structure prediction (PSP) problem is to find a three dimensional native structure that has the lowest free energy. In order to function properly, the protein has to fold into its native structure. Mis-folded proteins cause many critical diseases such as Alzheimer's disease, Cystic fibrosis, and Mad Cow disease. Knowledge about this native structure is of paramount importance and can have an enormous impact on the field of drug discovery. Not much is known about the folding process and the nature of the energy function is also very complex. For many decades, it has been considered one of the hardest problems in biology. *In vitro *laboratory methods like X-ray crystallography and Nuclear Magnetic Resonance (NMR) spectroscopy are very much slow and expensive. For these issues, many researchers from other fields are attracted to solve the problem using their own techniques [1,2].

Computational methods applied to PSP fall into three broad categories: *ab initio, homology modeling *and *protein threading*. The later two methods depend on the templates (or structures) of known proteins and are useful only when matching templates are found. Research in *ab initio *PSP has been instigated by the famous *Anfinsen's dogma*. In 1973 Nobel Prize Laureate Christian B. Anfinsen suggested that the native structure of a globular protein is determined only by its primary amino acid sequence [3]. The *ab initio *PSP can be viewed as a search problem, where one has to find a stable, unique, and kinetically accessible native structure from the space of all possible structures (also called conformations). The search space for this problem, even in the simplified models, contains an astronomically large number of conformations. Therefore, systematic search techniques are almost impractical since they perform exhaustive search and requires a huge amount of computational resources. In contrast, local search methods are normally very quick in finding good solutions, although they suffer from re-visitation and stagnation, and require good heuristics.

Performance of the computational methods also degrades when applied to the high resolution models that deal with real structures of proteins. This is due to three reasons: i) the unknown contributing factors of different forces to the energy functions, ii) protein models with atomic level details require huge computational effort, and iii) the space of possible conformations is very large and complex. For these reasons, the general paradigm of *de novo *PSP is to begin with the sampling of a large set of candidate (*decoy*) structures guided by a scoring function. In the final stage, the refinements are done to achieve the real structure. The simplified models, though lack many details, provide a realistic back-bone for the proteins and can be refined to get real structures [4].

Local search algorithms when applied to large proteins (sequence length around 200 monomers) suffer from a huge number of re-visitation and stagnation. To handle these issues, a number of techniques have been applied in the literature of PSP [5-7] that include tabu lists, adaptive measures, and various restart mechanisms. Similar approaches have also been used in other domains such as propositional satisfiability [8] and quadratic assignment problem [9]. Many of the algorithms apply random restarts or restart from the best local minimum [6,7]; which do not solve the problem in general.

### Our contribution

In this paper, we present a new algorithm for the simplified protein structure prediction problem. During the search, our method selects the best candidate in each iteration, but memorizes the second best conformations that are generated but not selected or explored (called elite conformations) at each iteration. Whenever the search faces stagnation, we select the best conformation from this elite set and continue search from there. This retreat helps the search diverge. Similar techniques have been used in the systematic search techniques like A* search, but they require a huge amount of memory to store the unexplored frontier. We maintain only a small set of previously generated conformations by discarding conformations with similar fitness. It reduces the memory requirement and provides a mechanism to go back to earlier conformations with lower fitness value but with potential to lead towards better search regions. We also propose a new non-isomorphic encoding that reduce the non-unique or isomorphic conformations from the search space and makes the similarity matching of the conformations efficient. These isomorphic conformations are essentially same and show differences only because of the translational and rotational symmetry. We applied this encoding in our algorithm along with the long term memory of local minima proposed in [10]. Experimental results show that our algorithm significantly outperforms the state-of-the-art algorithms on standard benchmark proteins using Hydrophobic-Polar(HP) energy model and Face Centered Cubic (FCC) lattice.

### Related work

Lau and Dill [1] proposed a simplified HP energy model for protein structure prediction problem. It is proved to be a hard combinatorial problem [11]. Due to the complexity, several techniques and their hybridizations have been applied to solve the problem. The similarity with the thermodynamic nature of the protein folding allured the researchers to apply simulated annealing [12,13]. Genetic algorithms were first applied to solve this problem by Unger and Moult [14]. The basic genetic algorithm was subsequently improved by many researchers [15-17].

Yue and Dill [18] applied constraint based approaches for the first time and developed the Constraint Based Hydrophobic Core Construction (CHCC) algorithm. Their method had several pitfalls: CHCC could only support the HP model and failed to report degeneracy or non-unique structures for several protein sequences. The research group of Rolf Backofen developed a Constrained-based Protein Structure Prediction (CPSP) tool [19], which provided solutions to these problems. However, CPSP tool depends on pre-calculated cores and does not converge for larger protein sequences. Palu et al. [20] developed COLA solver using highly optimized constraints and propagators to obtain satisfactory results on small and medium-sized instances (*length *< 80). Lesh et al. [5] provided a novel set of transformations called *pull moves *extendible to any lattice. Both Lesh et al. [5] and Blazewicz et al. [21] implemented tabu search meta-heuristics in-dependent of each other.

Hybrid techniques that combine the power of different strategies provided better results. Using the pull moves, Klau et al. [22] proposed an interactive optimization framework called Human Guided Simple Search (HuGS). Using the same pull move set, Ullah et al. [23] proposed a two-stage optimization approach. Furthermore, Ullah et al. [24] combined local search and constraint programming approaches. They introduced a protein folding simulation procedure on FCC lattice and employed the COLA solver [20] to generate neighborhood states for a simulated annealing based local search. They used MJ matrices with 20 × 20 amino acid pairwise interactions. They tested their approaches on some real proteins (*length *< 80) from the Protein Data Bank (PDB). Jiang et al. [25] combined tabu search strategy (GTS) with genetic algorithms in the two-dimensional HP Model.

Cebrian et al. [26] used tabu search to find 3D structures of Harvard instances [27] on FCC lattices for the first time. In their subsequent work, Dotu et al. [6,7] applied Large Neighborhood Search (LNS) to further optimize the results found in [26]. They also improved the tabu search by adopting a new neighborhood selection technique [7]. Both of their methods are implemented in COMET. Shatabda et al. [10] proposed a memory based approach on top of the algorithm proposed by Dotu et al. [7] and improved the results on the FCC lattice and HP energy model. Other methods (such as Simulated Annealing [12], Ant Colony Optimization (ACO) [28], and Extremal Optimization [29]) are also found in the literature.

## Materials and methods

Proteins are polymers of amino acid monomers. In a simplified model, all monomers have an equal size and all bonds are of an equal length. Each amino acid monomer is represented by a single point and its position is restricted to a three dimensional lattice. A simplified energy function is used in calculating the energy of a conformation. The given amino acid sequence fits into a fixed lattice, where every two consecutive monomers in the sequence are also neighbor on the lattice (called the *chain constraint*) and two monomers can not occupy the same lattice point (called the *self avoiding constraint*).

### FCC lattice

The Face Centered Cubic (FCC) lattice is preferred over other lattices since it has the highest packing density [30] for spheres of equal size, and provides the highest degree of freedom for placing an amino acid monomer. Thus, it provides a realistic discrete mapping for proteins. The FCC lattice is generated by the following basis vectors: $\vec{v_{1}}=\left(1,\;1,\;0\right)$, $\vec{v_{2}}=\left(-1,\;-1,\;0\right)$, $\vec{v_{3}}=\;\left(-1,\;1,\;0\right)$, $\vec{v_{4}}=\left(1,\;-1,\;0\right)$, $\vec{v_{5}}=\left(0,\;1,\;1\right)$, $\vec{v_{6}}=\left(0,\;1,\;-1\right)$, $\vec{v_{7}}=\left(0,\;-1,\;-1\right)$, $\vec{v_{8}}=\left(0,\;-1,\;1\right)$, $\vec{v_{9}}=\left(1,\;0,\;1\right)$, $\vec{v_{10}}=\left(-1,\;0,\;1\right)$, $\vec{v_{11}}=\left(-1,\;0,\;-1\right)$,$\vec{v_{12}}=\left(1,\;0,\;-1\right)$. Two lattice points *p*, q∈L are said to be in *contact *or *neighbors *of each other, if $q=p+\vec{v_{i}}$ for some vector $\vec{v_{i}}$ in the basis of lattice L .

### HP energy model

The Hydrophobic-Polar (HP) energy model was proposed by Lau and Dill [1]. In this model, all the amino acids are divided into two groups: hydrophobic H (*Gly, Ala, Pro, Val, Leu, Ile, Met, Phe, Tyr, Trp*); and hydrophilic or polar P (*Ser, Thr, Cys, Asn, Gln, Lys, His, Arg, Asp, Glu*). The given amino acid sequence of a protein is represented as a string s of the alphabet {*H, P*}. The free energy calculation for the HP model, shown in (1), counts only the energy interactions between two non-consecutive amino acid monomers.

(1) $$E=\underset{i,j:i+1<j}{\sum }c_{ij}.e_{ij}$$

where *c_ij_*= 1 only if two monomers *i *and *j *are neighbors (or in contact) on the lattice and 0 otherwise. The other term, *e_ij_*is calculated depending on the type of amino acids: *e_ij_*= -1 if *s_i_*= *s_j_*= *H *and 0 otherwise. Minimizing the summation in (1) is equivalent to maximizing the number of non-consecutive H-H contacts. Several other variants of HP-model [31] exist in the literature.

Using the HP energy model together with the FCC lattice, the simplified PSP problem is defined as: given a sequence *s *of length *n*, find a self avoiding walk *p*_1 _⋯ *p_n_*on the lattice such that the energy defined by (1) is minimized.

#### Local search framework

The local search framework was originally proposed in [7]. The algorithm is similar to that of the procedure *localSearch *() presented in Table 1 except in Lines 6, 9-10 and 14. It depends on a structured randomized initialization method and maintains a simple tabu list to prevent recently used moves. In the framework, moves involving single monomer are only allowed. For any given conformation *c *and a sequence position *i*, a *move*(*i, p, c*) that moves an amino acid *i *to a new position *p *is allowed, if (i) *p *is free and is in contact with both amino acids at positions *i *- 1 and *i *+ 1, and (ii) *i *is not in the tabu list. The length of the tabu list takes a random value from [4, *n*/4], where *n *is the length of the sequence. The move can be applied to either *H *or *P *type of amino acid at each iteration. The fitness function minimizes the summation of HH-distances for all non-consecutive pairs of H-monomers. The fitness function can be formally defined as the following:

**Table 1:** Local Setach Framework.

Procedure localSearch()		Procedure selectMove()	
**1**	initialize()	**1**	**while ***moveList.notEmpty() ***do**
**2**	initializeTabu()	**2**	*m *← getNextCandidate()
**3**	**while ***iteration *≤ *maxIteration ***do**	**3**	*c *← getConformation(*m*)
**4**	selectMonomerType()	**4**	*e *← getNonIsoEncoding(*s*)
**5**	generateMoves()	**5**	*b *← getPacked(*s*)
**6**	selectMove()	**6**	**if ***match(b, proximity) ***then**
**7**	performMove()	**7**	discard *m*
**8**	updateCosts()	**8**	**else**
**9**	**if ***local minima is detected ***then**	**9**	updateEliteSet()
**10**	storeLocalMinima()	**10**	return *m*
**11**	**end**	**11**	**end**
**12**	**if ***nonImprovingSteps *≥ *maxStable ***then**	**12**	**end**
**13**	initializeTabu()	**13**	**if ***moveList.empty*() **then**
**14**	selectFromEliteSet()	**14**	*no moves possible*
**15**	**end**	**15**	*nonImprovingSteps *← *maxStable *+ 1
**16**	**end**	**16**	**end**

(2) $$f\left(c\right)=\overset{n}{\underset{i,j:i+1<j}{\sum }}{\left(dv\left(i,j\right)\right)}^{2}\times \;\left(s_{i}=H,s_{j}=H\right)$$

where *dv*(*i, j*) = *d*(*i, j*)^2 ^-2 and *d*(*i, j*) = (*x_i_*-*x_j_*)^2 ^+ (*y_i_*- *y_j_*)^2 ^+ (*z_i_*- *z_j_*)^2^, i.e. square of the Euclidean distance between the *i*th and *j*th amino acids in the current conformation *c *of a sequence *s *of length *n*. The energy level of the structure is still determined by the HP energy value. The fitness function is used to drive the search only. The search algorithm periodically switches the type of the acid and selects the best move on a amino-acid which is not in the tabulist. In case of *P *moves, it selects a random move since a move of *P *type amino acid does not affect the fitness function. The search restarts from the previously found best solution whenever the fitness function is not improving for *maxStable *steps. The memory-based search in [10] extends this local search framework. It stores a proportion of the local minima encountered and whenever a move is selected, it generates the conformation and checks similarity with the stored local minima. If the generated conformation is within a given proximity of a stored local minimum, the conformation is discarded. Hamming distance is used as the similarity measure and *relative encoding *to represent the conformations.

Our algorithm is developed on top of the memory-based search. The pseudo-code for our algorithm is depicted in Table 1. Our algorithm differs from the memory-based approach in Line 14 of Procedure *localSearch*() where we select a conformation from the *elite set *at stagnation and in Line 9 of Procedure *selectMove*() where we store the prominent but not selected candidate conformations into the *elite set*. It also differs in the encoding of the representation of the conformations. We do that at Line 4 of Procedure *selectMove*() before matching it with stored local minima and at Line 10 of Procedure *localSearch*() while storing the local minimum. Rest of this section describes the detail of the procedures of our algorithm.

#### Elite conformations

In each iteration of a local search, a number of conformations are generated. However, only a few of them are explored in the next iterations. In the case of a single candidate search, only a single conformation, which is typically the best conformation according to the heuristic, is selected for the next iteration. In successive iterations, the search goes on by generating the neighbors of the selected conformations. The other potential conformations with good fitness values are never used as the search is greedy in nature. We call them *elite conformations*. These conformations, if explored ever, may lead to better search regions. Note that, in the systematic search techniques, these conformations are stored and explored. However, they require a huge amount of memory. Moreover, the selection in a systematic search like A* search depends on a heuristic function that requires the goal to be known beforehand. In our case, the optimal structure is totally unknown and we can not afford to store a huge number of conformations. In our algorithm, we store the second best conformations and explore them whenever the search faces stagnation.

### Store

We store the second best conformations in each iteration in a set called *elite set*. At each iteration, when a move is selected, we update this elite set of conformations. The pseudo-code for the *updateEliteSet*() procedure is given in the right side of Table 2. We use a priority queue sorted in the order of fitness value and iteration number to store the elite conformations. Before inserting a conformation into the priority queue, we check for similarity in the stored local minima list and store it only if no match is found.

**Table 2:** Pseudo-code for Elite Set Methods.

Procedure updateEliteSet()		Procedure selectFromEliteSet()	
**1**	*sb *← set of second best candidates	**1**	**while ***eliteSet.notEmpty*() **do**
**2**	**while ***sb.notEmpty*() **do**	**2**	*c *← *eliteSet*.getTopElement()
**3**	*m *← sb.getNextCandidate()	**3**	*e *← getNonIsoEncoding(*c*)
**4**	*c *← getConformation(*m*)	**4**	*b *← getPacked(*e*)
**5**	*e *← getNonIsoEncoding(*c*)	**5**	**if ***match(b, proximity) == false ***then**
**6**	*b *← getPacked(*e*)	**6**	*elitSet*.release()
**7**	**if ***match(b, proximity) == false ***then**	**7**	return *c*
**8**	*eliteSet*.push(c)	**8**	**end**
**9**	**end**	**9**	*eliteSet*.popElement()
**10**	**end**	**10**	**end**

### Explore

We select the top element from the priority queue whenever the search stagnates. The search then continues from the selected elite conformation. The search algorithm, guided by the fitness function defined in (2), quickly forms a compact hydrophobic core at the center of the conformation and the greedy search oscillates within the same region of the search space before it can improve the fitness function to break the core or to form some alternate core. The detailed nature of the search is discussed in [10]. The oscillating nature indicates that if we select a conformation from a region in the search space, then we can ignore the other conformations with the same or near fitness value and within the temporal locality. Every time an elite conformation is selected form the list, we do that by discarding a fixed proportion of the top elements from the list. This results in eliminating the conformations that are similar in fitness value and structure, and are also temporally proximate. This retreat effectively helps the search diverge. It also reduces the memory requirement for the priority queue used. The detailed pseudo-code of the method is given in the left side of Table 2. The method *elitSet.release*() at Line 6 releases the top elements from the elite set.

#### Non-isomorphic encoding

**Figure 1:**
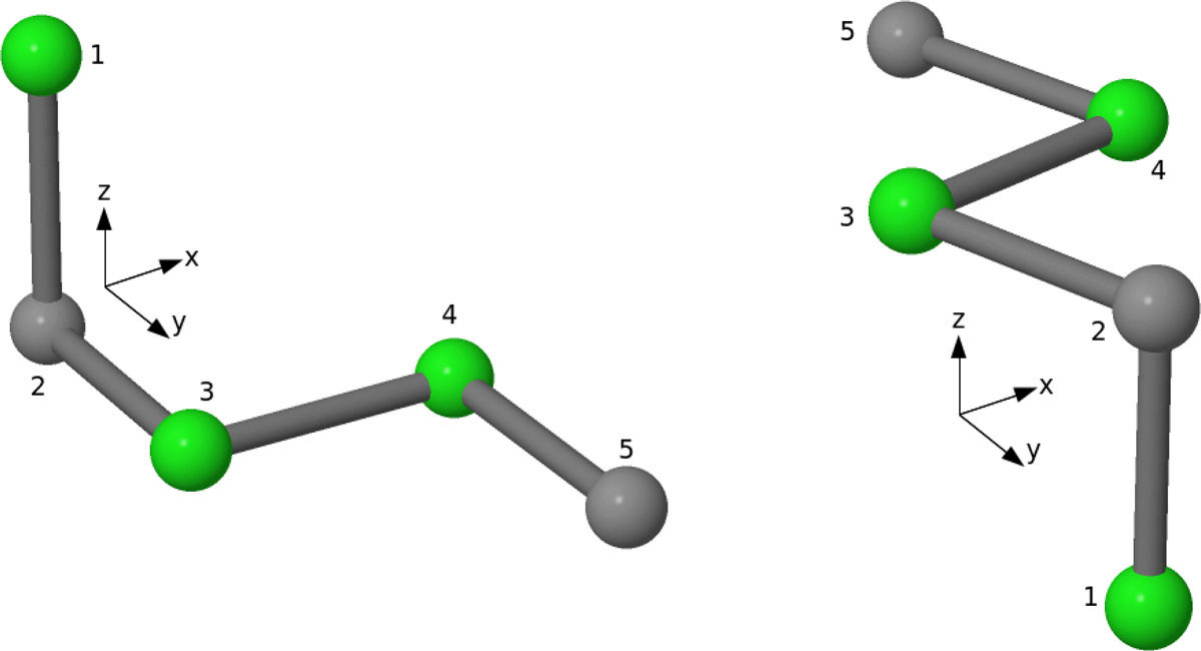
**Isomorphic Encoding**. Two identical structures in cubic lattice having different absolute encoding; structure in the left has the encoding "DSES", and the structure at right with encoding "UNEN", where D = Down U = Up, N = North, S = South, E = East and W = West.

Many techniques have been employed in the literature to represent the protein conformations. These representations allow the search to keep the candidate conformations updated and perform operations like similarity checking (memory-based algorithms) and crossover (genetic algorithms). The most obvious way to represent the conformations is to use Cartesian co-ordinates of the amino-acid monomers. However, such a representation contains translational symmetry, which can be solved if *absolute encoding *is used. Absolute encoding is found from the absolute direction vectors between the consecutive points in the amino-acid chain. The alphabet size of the absolute encoding depends on the lattice used. For the FCC lattice, the alphabet size is 12 since the number of basis vectors is 12. However, absolute encoding is not suitable when we check similarity between two conformations since it contains the problem of rotational symmetry. Two identical conformations with rotational symmetry are represented by different absolute encoding (see the example in Figure 1). This type of encoding is called *isomorphic encoding. Non-isomorphic encodings *provide a solution to this issue. Shatabda et al. [10] used the *relative encoding *proposed by Backofen et al. [32] in their algorithm. Their encoding scheme starts from a fixed direction and continues to update a base matrix throughout the chain. The efficiency of the algorithm thus depends of the dimension of the lattice. Moreover, a decoding algorithm is needed to get back the absolute encodings or the co-ordinate points. The computational complexity of their algorithm is *O*(*nl*^3^), where *n *is the number of absolute directions and *l *is the dimension of the lattice. The complexity of the decoding algorithm is also *O*(*nl*^3^). A non-isomorphic encoding was also proposed in [33] for cubic lattices that calculates the angles between two consecutive absolute direction vectors and en-codes the move sequence. This encoding also costs more as it requires computation of angles between the direction vectors.

In this paper, we propose a new non-isomorphic encoding, which is generic for any lattice and requires no separate decoding algorithm; the encoding itself maps to the absolute directions. Instead of relative angles, our algorithm depends on the relative occurrence of the absolute directions within the chain. It requires only *O*(*n*) time to encode. The pseudo-code of our algorithm is given in Table 3. This algorithm calculates the encoding on the fly. It starts with an empty *Map *and every time a new absolute direction is encountered in the sequence, it assigns the next available code to it. Once the mapping for all possible directions is found then the algorithm is just a simple lookup from the mapping array. In the results section, we show the effectiveness of our encoding scheme when applied to the memory-based search [10].

**Table 3:** Pseudo-code for Non-Isomorphic Encoding.

Procedure getNonIsoEncoding(s)	
**1**	initMap()
**2**	**for ***i *← 1 **to ***N ***do**
**3**	*absdir *= *c*.getAbsDir(*i*)
**4**	**if ***absdir is a new direction ***then**
**5**	*Map*[*absdir*] ← *dirCount*
**6**	*dirCount *+ +
**7**	**end**
**8**	*encoded*[*i*] = *Map*[*absdir*];
**9**	**end**
**10**	return *encoded*

## Results and discussion

We implemented our algorithm in C++ and ran experiments on the NICTA (http://www.nicta.com.au) cluster machine. The cluster has a number of machines each equipped with two 6-core CPUs (AMD Opteron @2.8 GHz, 3 MB L2/6 M L3 Cache) and 64 GB Memory, running Rocks OS (a Linux variant for cluster). We compared the performance of our algorithm to that of the tabu search by Dotu et al. [7] and the memory based approach proposed in [10]. Algorithms were run 50 times for each of the protein sequences. Each run was given 5 hours to finish. We could not compare our results with the Large Neighborhood Search (LNS) [7] since the COMET program exited with 'too much memory needed' error for the large-sized benchmark proteins that we have selected. We do not show results for small-sized Harvard instances (*length *= 48) or other smaller protein sequences since both algorithms reach near optimal conformations and the difference of the energy levels achieved for these proteins are relatively small.

### Results

We show results for two sets of benchmarks in Table 4. The first six proteins are also used by Dotu et al. [7]. The R instances (*length *= 200) are originally taken from [34] and the f180 instances (*length *= 200) are provided by Sebastian Will [7]. LS-New denotes our algorithm and LS-Mem denotes the memory-based approach in [10] and LS-Tabu de-notes the tabu search by Dotu et al. [7]. The best and average energy levels achieved are reported in Table 4. We set proximity measure to 3 and only 5% of the local minima was stored while *maxStable *was set to 100 for our algorithm. For other algorithms, we set the parameters as recommended by the authors. The best energy levels reported by Dotu et al. [7] are also shown under the column LNS. These results were produced by large neighborhood search. Optimal lower bounds for the minimum energy values for the proteins are also reported under the column '*E*_l_' generated by the CPSP tools [19]. Note that these values are obtained by using exhaustive search methods and are used only to evaluate how far our results are from them. The missing values indicate where no such bound was found and the values marked with * are the values for which the algorithm did not converge even after 24 hours of run.

**Table 4:** Experimental Results.

Protein			LS-New			LS-Mem			LS-Tabu		
				
**Seq**.	Length	*E*_l_	best	avg	best	avg	**R.I**.	best	avg	**R.I**.	LNS
R1	200	-384	-*359*	**-339**	-353	-326	22.41%	-332	-318	31.81%	-330
R2	200	-383	-*361*	**-343**	-351	-330	24.52%	-337	-324	32.20%	-333
R3	200	-385	-*354*	**-340**	-352	-330	18.18%	-339	-323	27.41%	-334
f180_1	180	-378*	-*361*	**-341**	-360	-334	15.90%	-338	-327	27.45%	-293
f180_2	180	-381*	-*368*	**-350**	-362	-340	24.39%	-345	-334	34.02%	-312
f180_3	180	-378	-*365*	**-355**	-357	-343	34.28%	-352	-339	41.02%	-313

3no6	229	-455	-*419*	**-397**	-400	-375	27.50%	-390	-373	29.26%	-
3mr7	189	-355	-*320*	**-304**	-311	-292	19.04%	-301	-287	25%	-
3mse	179	-323	-*288*	**-271**	-278	-254	22.63%	-266	-249	29.72%	-
3mqz	215	-474	-*430*	**-404**	-415	-386	20.45%	-401	-383	23.07%	-
3on7	279	?	-*514*	**-476**	-499	-463	-	-491	-461	-	-
3no3	258	-494	-*406*	**-376**	-397	-361	11.27%	-388	-359	12.59%	-

The best and average energy levels achieved and relative improvements of our algorithm over other algorithms for the R, f180 and instances taken from CASP.

We also used a second set of benchmark proteins derived from the famous Critical Assessment of Techniques for Protein Structure Prediction (CASP) competition (http://predictioncenter.org/casp9/targetlist.cgi). These proteins are of length 230 ± 50. Six protein sequences were randomly chosen from the target list. These sequences are then converted into HP sequences. Results for these six proteins are also given in Table 4 (lower part). The PDB ids for each of these proteins are also given. The parameter settings for these six proteins were also kept the same. LNS column contains no data for these six proteins since they were not used in [7].

### Analysis

From the average energy levels shown in bold-face in Table 4, it is clearly evident that, for all the twelve proteins, our algorithm significantly outperforms both of the algorithms. We performed statistical *t*-test for independent samples with 95% level of significance to verify the significant difference in performances. We report the new lowest energy levels (w.r.t. incomplete search methods) for all twelve proteins. These energy levels are shown in italic-faced font in Table 4.

### Relative improvement

In Table 4, we report the relative achievement in column 'R.I.'. Relative improvement of our approachis measured in terms of the difference with optimal bound of the energy level. This value is significant because it gets harder to find better conformations as the energy level of a protein sequence approaches the optimal. We define:

(3) $$\text{RelativeImprovement}=\frac{E_{\text{o}}-E_{\text{r}}}{E_{\text{l}}-E_{\text{r}}}\times 100\%$$

where *E*_o _is the average energy level achieved by our approach, *E*_r _is the average energy level achieved by the other approach, and *E*_l _is the optimal lower bound of the energy level. The missing values indicate the absence of any lower bound for the corresponding protein sequence. Similar measurements were also used in [10]. From the values reported in Table 4, we clearly see that our algorithm produces conformations that are significantly better in terms of the average energy level achieved.

### Search progress

**Figure 2:**
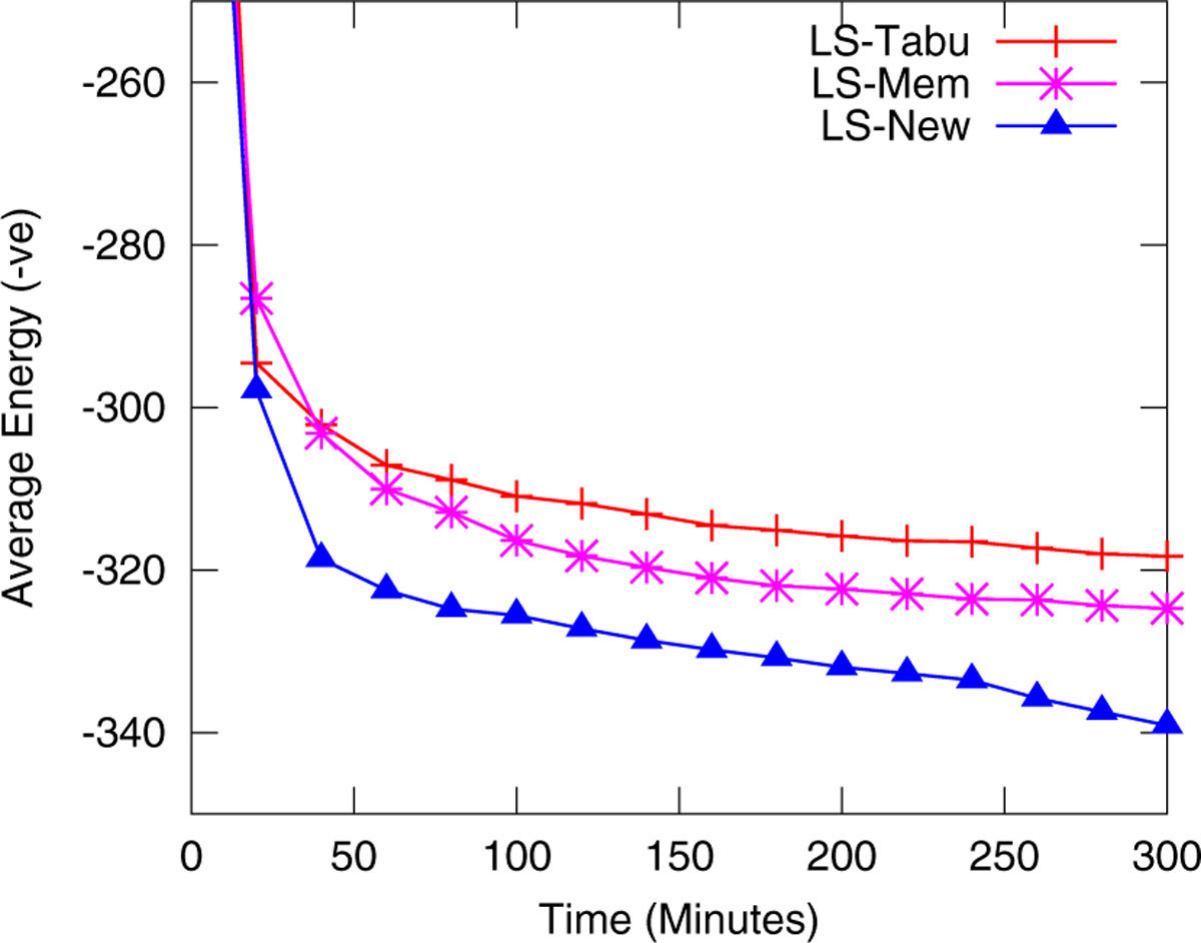
**Search Progress**. Search progress of three algorithms for Protein R1 over 300 minutes.

In Figure 2, we show search progress of three algorithms for the protein sequence R1. Average energy level by each of the algorithms for 50 runs are shown. All three algorithms achieve almost the same level of energy initially but as soon as the search makes progress, the tabu search and the memory-based search fail to overcome stagnation. It is clearly evident from the graph that our algorithm continues to improve in the stagnant situations and thus produces better results.

### Effect of the non-isomorphic encoding

The effects of the new non-isomorphic encoding of the protein conformations have been two-fold. Firstly, it resulted in the reduction of degeneracy, which is evident in the number of discarded conformations during the search. Secondly the efficient computation improved the runtime. In the memory-based approach proposed in [10], the authors used the relative encoding proposed in [32]. When applied with the memory-based algorithm proposed in [10], our new encoding resulted in more discards and less computation time, as shown in Table 5. The discarded conformations are the approximate measure of similar conformations encountered during the search. The experimental results for six proteins are shown in Table 5 for first one million iterations.

**Table 5:** Effect of Non-Isomorphic Encoding.

Protein	Our Encoding		Relative Encoding	
	
**Seq**.	runtime	# discards	runtime	# of discards
R1	28.33	354712	39.6	91664
R2	30.4	406572	42.6	91219
R3	25.74	475357	42.6	92765
f180_1	22.90	402738	35.4	103059
f180_2	27.34	317317	39.0	93814
f180_3	24.54	358326	37.8	89810

Comparison in runtime (in minutes) and the numbers of discards while using our non-isomorphic encoding and the relative encoding [32] for first 1 million iterations of the memory-based algorithm by Shatabda et al. [10].

## Conclusions

In this paper, we presented a local search algorithm for solving the protein structure prediction problem on FCC lattice using low resolution HP energy model. Experimental results shows that our algorithm outperforms the state-of-the art algorithms. We used a novel encoding scheme to represent the conformations along with a set of elite conformations to handle the stagnation of the local search. We believe that use of domain specific heuristics while selecting the conformations from the elite set can further improve the performance of the algorithm. In future, we wish to explore that and apply our techniques to higher resolutions and other energy models to see the effect. We wish to apply our techniques to other domains such as propositional satisfiability, vehicle routing. We believe the proposed encoding scheme will add efficiency to search techniques such as genetic algorithms.

## Competing interests

The authors declare that they have no competing interests.

## Authors' contributions

SS conceived the original idea of elite conformations and non-isomorphic encoding. All authors contributed significantly in the implementation, experimentation and writing of the manuscript and approved the final version.

## Declarations

The publication costs for this article were funded by the corresponding author's institution.

This article has been published as part of *BMC Bioinformatics *Volume 14 Supplement 2, 2013: Selected articles from the Eleventh Asia Pacific Bioinformatics Conference (APBC 2013): Bioinformatics. The full contents of the supplement are available online at http://www.biomedcentral.com/bmcbioinformatics/supplements/14/S2.
